# Monocyte-Derived Dendritic Cells from Patients with Dermatophytosis Restrict the Growth of *Trichophyton rubrum* and Induce CD4-T Cell Activation

**DOI:** 10.1371/journal.pone.0110879

**Published:** 2014-11-05

**Authors:** Karla Santiago, Gisele Facholi Bomfim, Paulo Ricardo Criado, Sandro Rogerio Almeida

**Affiliations:** 1 Departamento de Análises Clínicas e Toxicológicas, Faculdade de Ciências Farmacêuticas, Universidade de São Paulo, São Paulo, Brazil; 2 Departamento de Dermatologia, Faculdade de Medicina, Universidade de São Paulo, São Paulo, Brazil; Institut de Pharmacologie et de Biologie Structurale, France

## Abstract

Dermatophytes are the most common agents of superficial mycoses that are caused by mold fungi. *Trichophyton rubrum* is the most common pathogen causing dermatophytosis. The immunology of dermatophytosis is currently poorly understood. Recently, our group investigated the interaction of *T. rubrum* conidia with peritoneal mouse macrophages. We found that macrophages phagocytose *T. rubrum* conidia resulted in a down-modulation of class II major histocompatibility complex (MHC) antigens and in the expression of co-stimulatory molecules. Furthermore, it induced the production of IL-10, and *T. rubrum* conidia differentiated into hyphae that grew and killed the macrophages after 8 hrs of culture. This work demonstrated that dendritic cells (DCs) and macrophages, from patients or normal individuals, avidly interact with pathogenic fungus *T. rubrum*. The dermatophyte has two major receptors on human monocyte-derived DC: DC-SIGN and mannose receptor. In contrast macrophage has only mannose receptor that participates in the phagocytosis or bound process. Another striking aspect of this study is that unlike macrophages that permit rapid growth of *T. rubrum*, human DC inhibited the growth and induces Th activation. The ability of DC from patients to interact and kill *T. rubrum* and to present Ags to T cells suggests that DC may play an important role in the host response to *T. rubrum* infection by coordinating the development of cellular immune response.

## Introduction

Dermatophytes are a group of fungi that have the capacity to invade keratinized tissue to produce infection denominate Dermatophytosis. Dermatophytosis are caused by mold fungi belonging to the genera *Trichophyton, Microsporum, and Epidermophyton* [1]. Among the human pathogenic dermatophytes, the anthropophilic species *Trichophyton rubrum* is clinically the most commonly observed [2]. Global prevalence of dermatomycoses is as high as 20% according to the World Health Organization and approximately 10% of the human population suffers of onychomycosis [3]. Severe dermatophytosis is often seen in AIDS patients [4] [5] indicating the importance of cellular immunity in the control of fungal growth. Disseminated deep dermatophytosis is a rare presentation of *T. rubrum* infection, which occurs exclusively in immunosuppressed patients [6].

The immunology of dermatophytosis is currently poorly understood. Some works have focused on T cell immunity against dermatophytes [7], [8]. It is now accepted that a cell-mediated immune (CMI) response is responsible for the control of infection by dermatophytes. On the other hand, susceptibility to chronic dermatophytosis is associated with atopy and with immediate type hypersensitivity [9].

The understanding of innate immunity against fungi has experienced great progress throughout the past few years with the discovery of Toll-like receptors and glucan receptors such as dectin-1 and dectin-2 [9] [10]. The interaction of fungi with these receptors has been contributing to the better comprehension of the innate response. Professional phagocytes, consisting of neutrophils, macrophages and dendritic cells, have an essential role in the initiation of the specific immune response [11].

Innate immunity is instrumental for the development of adaptive cell-mediated immune responses controlling mycotic infections or for disease progression. It participates in conferring phagocytic cells their ability to ingest and/or inhibit fungal growth, as well as in triggering an acute inflammatory reaction, or in presenting fungal antigens to T cells [12], [13]. These cells could phagocytose fungal cells, initiate antigen presentation, as well as secrete key cytokines such as tumor necrosis factor-α (TNF-α) and interleukin (IL)-12 [14], chemokines and other molecules that initiate the inflammatory reaction and modulate the infection [15].

Dendritic cells (DCs) are antigen-presenting cells able to “link” between the innate and adaptive immune response, being the only ones to migrate to secondary lymphoid organs to present antigen to naive T cells to become antigen presenting cells “Professional” [16]. Besides being efficient at capturing antigen, are able to process them and present their peptides via MHC-II to T lymphocytes “naïve” [17].

Dendritic cells are located at sites of antigen exposure as the mucosa and peripheral tissues. The precursors of these cells originate in the bone marrow and migrate to constantly various tissues such as skin (where they are known as Langerhans cells), the gastrointestinal, respiratory, blood and lymph. In peripheral tissues, these cells are inefficient in antigen presentation and activation of T cells, because they have immature phenotype [18]. Immature dendritic cells have low expression of co-stimulatory molecules and molecules of MHC class I and II but have large phagocytic capacity.

Our group investigated the interaction of *T. rubrum* conidia with peritoneal mouse macrophages [19]. We found that macrophages phagocytose *T. rubrum* conidia and that this process is inhibited by *T. rubrum* exoantigens or mannan. We also found that the phagocytosis of *T. rubrum* has functional consequences for macrophages since it resulted in a down-modulation of MHC class II and in the expression of co-stimulatory molecules. Furthermore, it induced the production of IL-10, a potent anti-inflammatory cytokine. Moreover, the ingested *T. rubrum* conidia differentiated into hyphae that grew and killed the macrophages after 8 hrs of culture. These results indicated that fungal was able to inhibit macrophage functions or to induce suppressive cytokines that could favor fungal evasion from host responses. The aim of this work was evaluate the interaction of macrophages and dendritic cells from patients or control individuals with conidia of *T. rubrum*.

## Materials and Methods

### Patients

Ten patients (7 men and 3 women; age range: 40–70 years) with clinical diagnoses of dermathophytosis, attended at the Department of Dermatology, Medical School of the University of São Paulo, were included in the present study. All diagnoses were confirmed by direct mycological examination and culture. In all cases, *Trichophyton rubrum* fungus was isolated. Healthy individuals (10 individuals: 7 men and 3 women; age range: 35–75 years) with no history of dermathophytosis were used as controls.

### Ethics Statement

This study was approved by Ethical Committee of Faculty of Pharmacy and Medical School – University of Sao Paulo. The participants provide their written informed consent to participate in this study.

### Preparation of human macrophage

Human monocytes were purified from patients or control. After partial purification via Ficoll-Hypaque centrifugation, monocytes were separated from lymphocytes by positive selection with anti-CD14. For positive selection of CD14^+^ monocytes, PBMCs were mixed with antihuman CD14 antibody-conjugated microbeads (Miltenyi Biotec, Auburn, CA, USA). CD14^+^ monocytes were separated from other cells using a magnetic separation column under a magnetic field (Miltenyi Biotec, Auburn, CA, USA) according to the manufacturer instructions and washed thoroughly to remove any non-specific binding to the beads. CD14^+^ cells were then eluted in PBS containing fetal-calf serum (FCS) (2%). Macrophages were obtained by culture of monocytes at 1×10^6^/ml in Teflon beakers in RPMI 1640 medium containing 15% human serum, 10 µg/ml gentamicin (Sigma-Aldrich) and 100 U/ml penicillin and 100 µg/ml streptomycin (Sigma-Aldrich) for 5–7 days.

### Generation of DCs

PBMCs were isolated by gradient centrifugation over Ficoll-Hypaque (400×g, 30 min). The phase containing white cells was removed and washed in RPMI-1640. CD14^+^ monocytes were separated from other cells using a magnetic separation column under a magnetic field (Miltenyi Biotec, Auburn, CA, USA) as described above. Isolated CD14^+^ cells were cultured in presence of granulocyte-macrophage colony-stimulating factor (GM-CSF) and IL-4 (50 and 1 ng/ml, respectively; 7 days). Cultures were fed every 2 days with GM-CSF and IL-4. DCs thus obtained were more than 95% as assessed by flow-cytometry analysis.

### Conidia of *T. rubrum*

In order to obtain large numbers of conidia, fungal fragments from Sabouraud medium cultures were scraped off the agar and incubated in Potato-broth medium (50 g of potatoes, 5 g of glucose (MERCK – Germany) and 500 mL of distilled water) at 25°C under constant rotation for 4 days. The fungal suspension was then filtered through a sterile micropore Whatman #1 filter to remove hyphae fragments but not microconidia. The conidia were washed with PBS and counted in a hemocytometer.

### Exoantigens from *T. rubrum*

Exoantigens from *T. rubrum* were obtained from fungal fragments that were scraped from Sabouraud medium agar and incubated in Sabouraud medium broth under constant rotation for 7 days, at room temperature. The fungal suspension was then filtered to remove hyphae fragments and/or microconidia. Protein contents were determined by the Bradford method.

### Interaction of macrophages and dendritic cell with conidia

1×10^6^ macrophages and dendritic cells were layered on glass coverslips for 1 h to allow for adherence. The glass attached macrophages and dendritic cells were rinsed in PBS and placed in 24-well plates and incubated with 1 mL complete RPMI-1640 medium containing conidia (rate conidia/cell = 3∶1) for 4, 8, and 12 h at 37°C in a 5% CO_2_ atmosphere. The glass coverslips were then washed with PBS and stained with Giemsa. To assess the involvement of mannose and DC-SIGN receptors on phagocytosis, assays were performed incubating anti-CD206 or anti-CD209 or both for 30 minutes before the addition of conidia. A dose response experiment using 1, 10, 20 and 50 µg/ml of antibody was realized. As control was used the purified mouse IgG1 kappa isotype to anti-CD206, and purified mouse IgG2b kappa isotype control to anti-CD209. For inhibition studies, macrophages and dendritic cells were incubated with different concentrations of exoantigen (5, 10, 15 mg/ml) for 30 min before the addition of *T. rubrum*.

Subsequently, after 12 h of phagocytosis, the coverslips were washed with 1X PBS to remove non- phagocytosed or bound conidia and stained with Giemsa (MERCK - Germany). After staining, the coverslips were fixed with Entellan (MERCK - Germany) and subsequently analyzed by standard optical microscope (400x and 1000x).

The Phagocytic Index (PI) is the percentage of phagocytic cells in relation to the total number of cells multiplied by the mean number of internalized or bound particles.

### Citotoxicity assays

Cell viability of *T. rubrum*-infected macrophages and dendritic cells was determined by measuring the release of LDH. Briefly, cells suspensions containing 1×10^6^ macrophages and dendritic cells were layered onto 96-well tissue culture microplates (Corning costar Co., Cambridge, MA) for 1 h to allow adherence. The attached macrophages and dendritic cells were rinsed in PBS and incubated with 1 mL complete RPMI-1640 medium containing conidia at 1∶3 ratio in 100 µL in RPMI complete medium for 4, 8, 12 and 16 h at 37°C in a 5% CO_2_ atmosphere. The supernatant was collected for lactate dehydrogenase (LDH) determination. Briefly, 50 µL of samples were mixed with color reagent LDH Liquiform (Labtest Diagnostic) and the absorbance was measured at 320 nm using a multispectrophotometer (Cobas Mira Plus – Roche). The concentration of LDH in samples was then determined using a standard curves generated by the serial dilution of LDH.

### Cytokine measurements

The levels of TNF-α, IL-12 and IL-10 in the supernatants of cells cultures were determined using a sandwich ELISA kit according to manufacturer’s instructions. Cells were incubated with conidia as previously described and after 12 h the supernatants were harvested and stored at –70°C for cytokine quantification. TNF-α, IL-12 and IL-10 were quantified in the supernatants by enzyme-linked immunosorbent assay (ELISA) (R&D), according to the manufacturer’s instructions. Cytokine activity was determined employing curves with serial dilutions of mouse recombinant TNF-α, IL-12 and IL-10. The results were expressed as a means obtained from two experiments +/− standard deviation (S.D.).

### Assays of Ag presentation

DCs were prepared as described above and incubating (24 h) with conidia of *T. rubrum* (rate conidia/cell = 3∶1). Afterwards, DCs were plated (2×10^4^ cells/well, triplicate) on a 96-well flat-bottom plate. Autologous CD4+ T cells were purified from non-adherent PBMCs using positive separation with anti-human CD4 antibody-conjugated microbeads and added (2×10^5^ cells/well). Proliferation of CD4 T cells was evaluated after 5 days of culture by measuring [^3^H]-thymidine uptake during the last 16 h of the assay. As negative control was used only CD4 T cells. The supernatant of this assay was harvest and assayed for IFN-γ, IL-4 and IL-10 by enzyme-linked immunosorbent assay (ELISA) according to the manufacturer instructions (R&D).

### Statistical analysis

Statistical comparisons were made by analysis of variance (ANOVA) and by the Tukey-Kramer test. All values were reported as the mean +/− standard error deviation of the means.

## Results

### Outcome of T. rubrum infection in macrophages and dendritic cells

**Figure 1:**
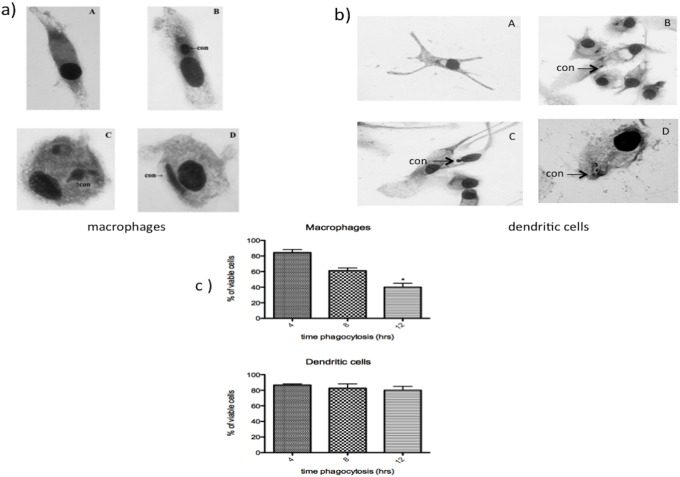
Outcome of *T.rubrum* infection in macrophages and dendritic cells. Cells layered on coverslips inserted in 24-well tissue culture microplates and incubated with conidia for different time intervals. (a) The coverslips were washed and stained with Giemsa for micrographs. (A) Macrophages and dendritic cells cultured without conidia for 10 h; (B), macrophage infected with conidia for 4 h; (C) 8 h and (D) 12 h. (b) Fungal citotoxicity was determined by LDH assay. The arrow indicates the presence of conidea. *p<0.05 as compared with macrophages and dendritic cell cultivated with *T.rubrum* conidia after 4 h of interaction.

**Figure 2:**
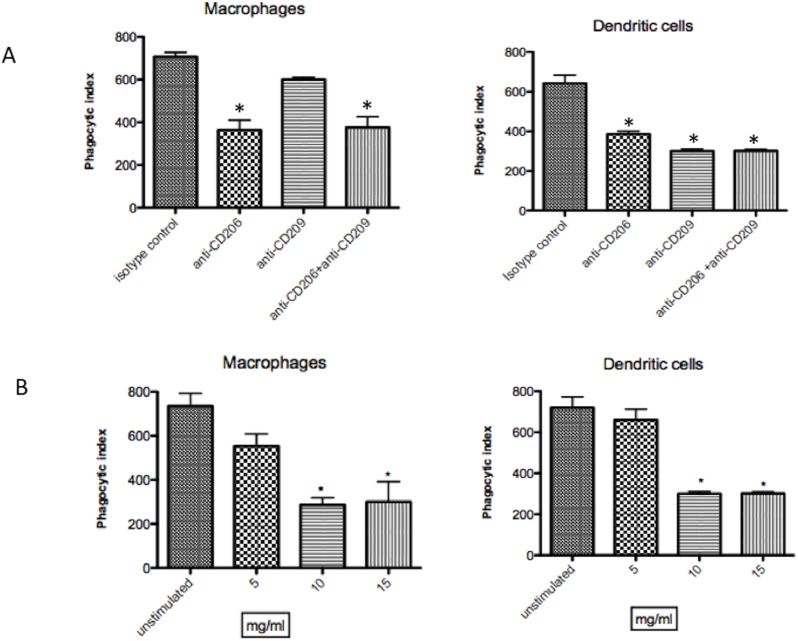
Phagocytic index of *T. rubrum* in presence inhibitors of mannose and DC-SIGN receptors or both. Macrophages and dendritic cells was incubated with 1, 10, 20 and 50 µg/ml of antibody and co-cultured with *T. rubrum* conidia at a ratio of 1∶3. After 12 h the cells were fixed and stained with Giemsa. Internalized fungal cells were visualized by light microscopy and the phagocytic index determined (A). As control was used the purified mouse IgG1 kappa isotype to anti-CD206, and purified mouse IgG2b kappa isotype control to anti-CD209. The graph represents the inhibition using 10 µg/ml of antiboby, that was determined previously as the optimal dose (A). Effect of exoantigen (B) on phagocytosis of *T. rubrum* conidia by macrophages and dendritic cells. Macrophages and dendritic cells were incubated with exoantigen at different concentrations for 30 min before the addition of *T. rubrum.* *p<0.05 when compared with macrophages or dendritic cells cultivated with isotype antibody or unstimulated (control).

We were interested in determining the fate of *T. rubrum* after its uptake by macrophages and dendritic cells from patients and normal individuos, since that was demostrate by our lab that macrophage from mice were susceptible to grows of fungus. For this, human macrophages and dendritic cells were infected with conidia and after different intervals of time fixed, stained and examined under light microscopy. The analyze of microscopy showed that after interaction of *T. rubrum* conidia with patients macrophages, the fungus differentiated into hyphae after 8 hrs of incubation (Figure 1a). In contrast, the conidea was not be able to differentiated in hyphae in the presence of DCs from patients (Figure 1b). The same results was observed using cells from normal individuos (data not shown). Cytotoxicity assays revealed that macrophage viability decreased with time of culture. After 10 h of culture the majority of macrophages died as determined by LDH activity in macrophage supernatants (Figure 1c). On the other hand, dendritic cells were not affected by conidea, even after 10 hours of incubation remained viable (Figure 1c). In order to verify the participation of mannose and DC-SIGN receptors on phagocytosis, dendritic cells and monocyte derived from patients were incubated with anti-CD206 and anti-CD209 for 30 minutes. After this period, was added to culture, *T.rubrum* conidia and evaluated the phagocytic index. A decrease in the rate of phagocytic index of conidia by dendrtic cell was observed when blocked by mannan (CD206) and DC-SIGN (CD209) receptors (Figure 2A). Horewer was observed inhibition of phagocytosis or bound by macrophages only when these cells were incubated with anti-CD206 (Figure 2A). Since exoantigens contain carbohydrates and fungal-derived carbohydrates that are rich in mannan, we incubated the macrophages and dendritic cells with exoantigens of *T. rubrum* 30 min after adding conidia. The presence of exoantigens during the phagocytic test inhibited in a dose-dependent manner the phagocytic index (Figure 2B).

### Production of cytokines by macrophages and human DCs exposed to *T. rubrum in vitro*

**Figure 3:**
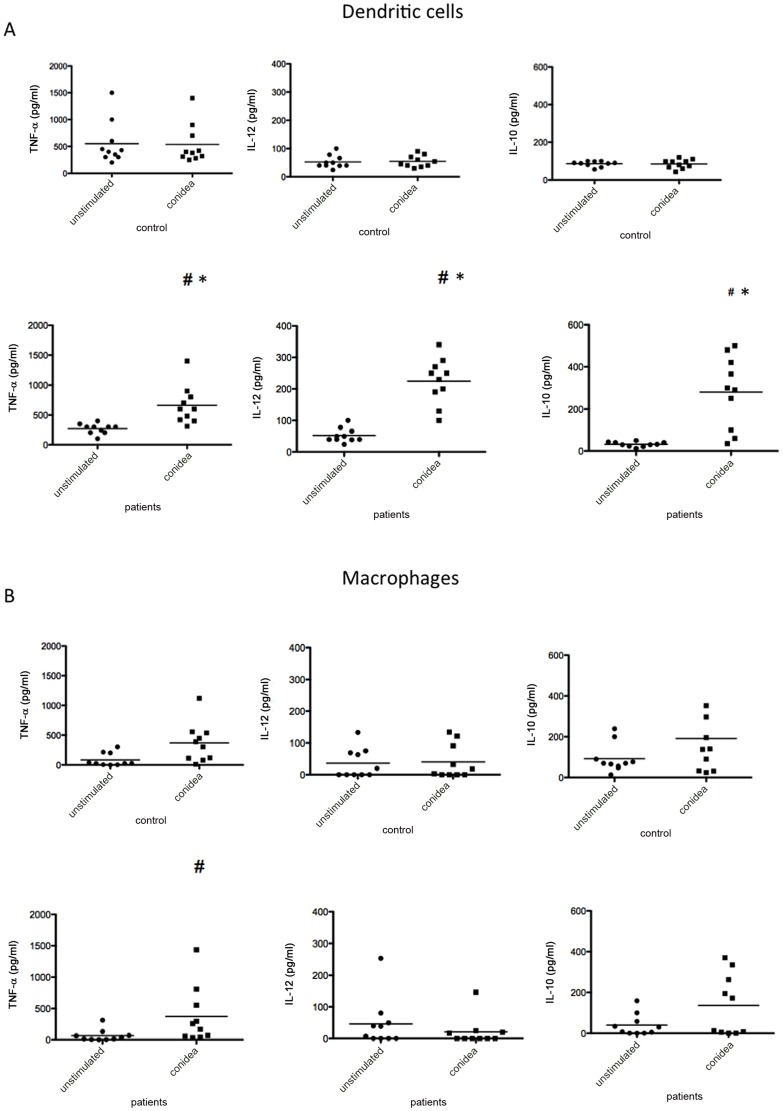
Influence of *T. rubrum* conidia on the release of TNF-α, IL-12 and IL-10 by dendritic cells (A) and macrophages (B). After 12 h the supernatants were harvested, then TNF-α, IL-12 and IL-10 quantified by ELISA. Results are representative of three independent experiments. Each dot correspond to an individual. #p<0.05 when compared with cells unstimulated, *p<0.05 when compared with macrophages.

In order to assess the pattern of cytokine production by macrophages and DCs upon interaction of the fungus, macrophages and DCs from patients and normal individuals were exposed to the fungus. Cytokines were determined by ELISA. Cytokine analysis showed that only basal levels of IL-12, IL-10, and TNF-α were produced by cell from controls. However, high levels of IL-12, IL-10, and TNF-α were produced when DCs from patients were stimulated with conidia when compared with DC controls and macrophages (Figure 3A). On the other hand, the macrophages from patients produced IL-10 and TNF-α, but not IL-12 (Figure 3B).

### Proliferation of T cells

**Figure 4:**
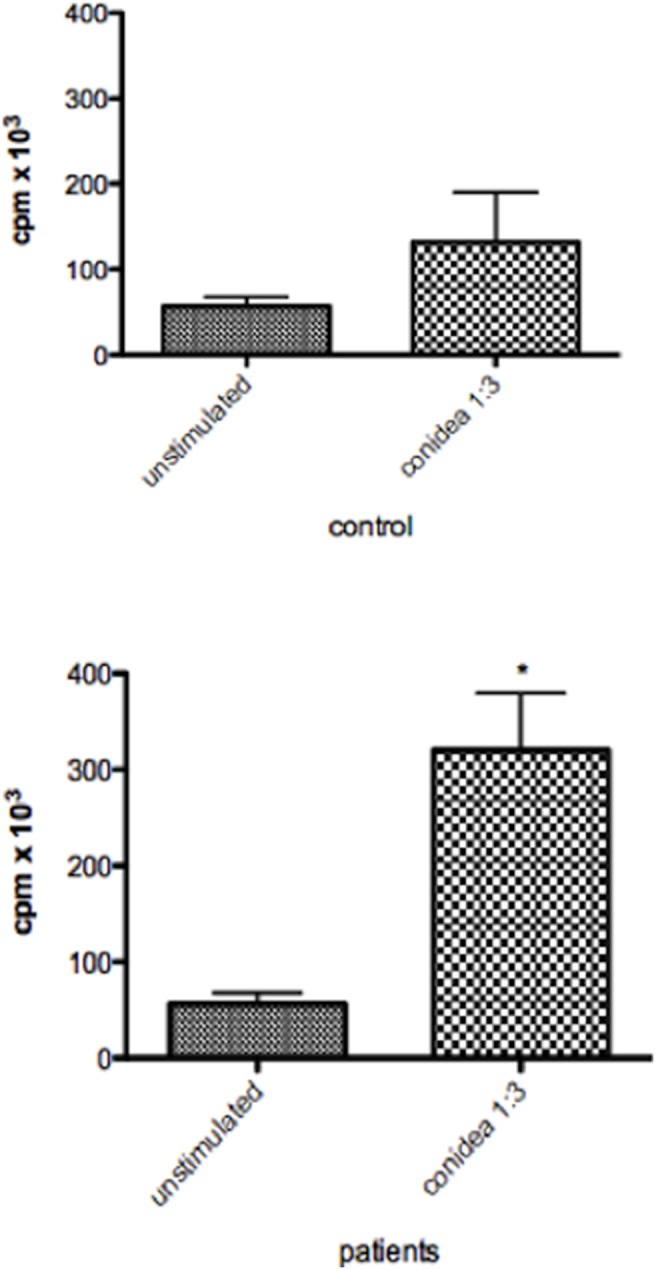
Proliferation of CD4+ T-cell in presence of *T. rubrum* conidea-pulsed DCs from patients or non-infected individuals. On day 7, DCs were pulsed with or without conidia of *T.rubrum* (rate conidia/cell = 3∶1) by 24 hr. After, pulsed DCs were plated at 2×10^4^ in triplicate wells of a 96-well flat-bottom plate. Autologous CD4+ T-cell were added at 2×10^5^ cells/well. Proliferation of CD4 T-cell was evaluated after 5 days by measuring [^3^H]thymidine uptake and results were expressed as counts per minutes (CPM). The results represent the mean ± SD of three independents experiments, where the blood was collected from each study subjects (10 patients and 10 controls). As negative control was used only CD4+ T-cell and the result of proliferation was <100 cpm. Each dot correspond to an individual. *p<0.05 was considerate significant.

**Figure 5:**
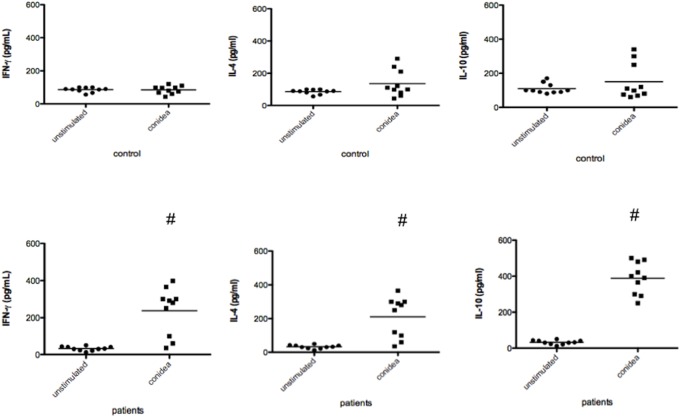
Cytokine production by CD4+ T-cell in presence of DCs and *T. rubrum* conidia (rate conidia/cell = 3∶1). After 5 days of proliferation the supernatant was harvested and cytokine determined. The results represent the mean ± SD of three independents experiments, where the blood was collected from each study subjects (10 patients and 10 controls). As negative control was used only CD4+ T-cell and the result of cytokine production always <20 pg/ml. Each dot correspond to an individual. *p<0.05 was considerate significant.

As showed above that DCs were resistant to death by conidia, we assess whether the antigen presenting properties were preserved. DCs were prepared as described above and incubating (24 h) with conidia of *T. rubrum* (rate conidia/cell = 3∶1). The results showed that DCs incubated with conidea, were able to process and present antigen from the fungus and induce autologous T-cell proliferation in patients, but not in healthy non-immune individuals (Figure 4). The results indicate the specifity of the response, because T-cells from healthy individual did not proliferate in presence of dendritic cells. To analize the pattern of cytokine production by the CD4 cells, IL-4, IL-10 and IFN-γ were quantified. The results showed production of all cytokine tested, indicating that dendritic cells in presence of *T. rubrum* was not able to induce the predominance of Th1 (IFN-γ) neitheir Th2 (IL-10 and IL-4) response (Figure 5).

## Discussion

Recently we found that *T. rubrum* phagocytosis has functional consequences for macrophages since phagocytosis of *T. rubrum* resulted in down-modulation of MHC-II and co-stimulatory molecules expression and conidia differentiated in hyphae that grew and killed the macrophages [19]. These results indicate that fungal cells are able to inhibit macrophages functions that could favor fungal evasion from host responses. Here we determine if marcophages or dendritic cells from patients and control individuos could phagocyte and kill the *T. rubrum*.

DCs are the most potent APC of the immune system and are vital for the initiation of primary T cell-mediated immune responses that are the hallmark of CMI [20]. As host defense against *T. rubrum* requires the induction of CMI, we sought to determine a role for DCs and macrophages in this process. The data presented herein demonstrate that human DCs and macrophages avidly ingest the pathogenic fungus *T. rubrum*. Our findings clearly show that *T. rubrum* has two major receptors on human monocyte-derived DC – DC-SIGN and MR. In contrast macrophages has only MR receptor that participates in the phagocytosis or bound process. DC-SIGN is expressed at sites in the skin (dermis) where *T. rubrum* is known to enter the host. Therefore, DC-SIGN-positive DCs might, trough these C-type lectin receptors, form the first encounter with these pathogens and the host immune system and, after antigen presentation, initiate a cellular response [21] [22] [23]. The results also shown that others receptors could participate in the bind of *T.rubrum* with macrophages or dendritic cells.

Recentily, it have been demonstrated the collaboration between Pattern Recognition Receptors (PRRs) such as C-type lectin and Toll-like receptors in initiates optimal antifungal responses. The work demostrated that *Fonsecaea pedrosoi*, which causes chromoblastomycoses, was shown to be recognized by CLRs, but not TLRs, and this resulted in defective inflammatory responses and susceptibility to infection. Moreover, exogenous administration of TLR agonists restored protective inflammatory responses and led to clearance of the infection *in vivo* [24].

Another striking aspect of this study is that unlike macrophages that permit rapid intracellular growth of *T. rubrum*, human DCs inhibited the intracellular growth. In a previous study [19], we demonstrated that macrophages were not able to kill and digest internalized *T. rubrum*. Thus, it is curious that hyph generated from conidia was quite restricted in macrophages, we hypothesize that those conidia that were successful in this enterprise were able to modulate the phagosome, possibly by preventing phagolysosomal fusion. Further studies are required to address this hypothesis. Newman et *al.* 2011 demonstrate that macrophage provide a permissive environment for the transformation of conidia into yeasts of *Histoplasma capsulatum*. In contrast, conidia ingested by DCs are severely restricted in their ability to transform into yeasts compared to macrophages [25].

Since DCs are the main antigen presenting cells of the immune system, we analized with conidea residing in DCs are degraded and antigens processed for presentation to T cells. Ours results showed that DCs from patients were able to present antigens and activate and mix of Th1/Th2 response with production of IL-4, IL-10 and IFN-γ.

The ability of DCs to kill *T. rubrum* and to present Ags to T cells suggests that DCs may play an important role in the host response to *T. rubrum* infection by coordinating the development of CMI. Current efforts are directed toward delineating the mechanism by which human DCs inhibit the intracellular growth of conidea.

## References

[1] Weitzman I, Summerbell RC (1995) The dermatophytes. Clin Microbiol Rev 8: 240–259. 7621400 10.1128/cmr.8.2.240 PMC172857

[2] Seebacher C, Bouchara JP, Mignon B (2008) Updates on the epidemiology of dermatophyte infections. Mycopathologia 166: 335–352. 18478365 10.1007/s11046-008-9100-9

[3] Summerbell RC (1997) Epidemiology and ecology of onychomycosis. Dermatology 194 Suppl 132–36. 10.1159/000246182 9154399

[4] Gruseck E, Abeck D, Ring J (1993) Relapsing severe Trichophyton rubrum infections in an immunocompromised host: evidence of onychomycosis as a source of reinfection based on lectin typing. Mycoses 36: 275–278. 8114808 10.1111/j.1439-0507.1993.tb00765.x

[5] Korting HC, Blecher P, Stallmann D, Hamm G (1993) Dermatophytes on the feet of HIV-infected patients: frequency, species distribution, localization and antimicrobial susceptibility. Mycoses 36: 271–274. 8114807 10.1111/j.1439-0507.1993.tb00764.x

[6] Erbagci Z (2002) Deep dermatophytoses in association with atopy and diabetes mellitus: Majocchi's granuloma tricophyticum or dermatophytic pseudomycetoma? Mycopathologia 154: 163–169. 12206315 10.1023/a:1016328001146

[7] Dahl MV (1994) Dermatophytosis and the immune response. J Am Acad Dermatol 31: S34–41. 8077506 10.1016/s0190-9622(08)81265-0

[8] Woodfolk JA, Platts-Mills TA (1998) The immune response to dermatophytes. Res Immunol 149: 436–445 discussion 522–433. 9720961 10.1016/s0923-2494(98)80767-0

[9] Hoving JC, Wilson GJ, Brown GD (2014) Signalling C-Type lectin receptors, microbial recognition and immunity. Cell Microbiol 16: 185–194. 24330199 10.1111/cmi.12249 PMC4016756

[10] Romani L (2004) Immunity to fungal infections. Nat Rev Immunol 4: 1–23. 14661066 10.1038/nri1255

[11] Faro-Trindade I, Willment JA, Kerrigan AM, Redelinghuys P, Hadebe S, et al (2012) Characterisation of innate fungal recognition in the lung. PLoS One 7: e35675. 22536422 10.1371/journal.pone.0035675 PMC3334970

[12] Romani L (2011) Immunity to fungal infections. Nat Rev Immunol 11: 275–288. 21394104 10.1038/nri2939

[13] Romani L (1997) The T cell response against fungal infections. Curr Opin Immunol 9: 484–490. 9287178 10.1016/s0952-7915(97)80099-4

[14] Romani L, Puccetti P, Bistoni F (1997) Interleukin-12 in infectious diseases. Clin Microbiol Rev 10: 611–636. 9336665 10.1128/cmr.10.4.611 PMC172937

[15] Stevens DA, Walsh TJ, Bistoni F, Cenci E, Clemons KV, et al (1998) Cytokines and mycoses. Med Mycol 36 Suppl 1174–182. 9988506

[16] Montagnoli C, Bacci A, Bozza S, Gaziano R, Fiorucci S, et al (2001) The plasticity of dendritic cells at the host/fungal interface. Immunobiology 204: 582–589. 11846221 10.1078/0171-2985-00095

[17] Bozza S, Gaziano R, Spreca A, Bacci A, Montagnoli C, et al (2002) Dendritic cells transport conidia and hyphae of Aspergillus fumigatus from the airways to the draining lymph nodes and initiate disparate Th responses to the fungus. J Immunol 168: 1362–1371. 11801677 10.4049/jimmunol.168.3.1362

[18] Claudia M, Bacci A, Silvia B, Gaziano R, Spreca A, et al (2002) The interaction of fungi with dendritic cells: implications for Th immunity and vaccination. Curr Mol Med 2: 507–524. 12243244 10.2174/1566524023362203

[19] Campos MR, Russo M, Gomes E, Almeida SR (2006) Stimulation, inhibition and death of macrophages infected with Trichophyton rubrum. Microbes Infect 8: 372–379. 16293438 10.1016/j.micinf.2005.07.028

[20] Moulin V, Morgan ME, Eleveld-Trancikova D, Haanen JB, Wielders E, et al (2012) Targeting dendritic cells with antigen via dendritic cell-associated promoters. Cancer Gene Ther 19: 303–311. 22361816 10.1038/cgt.2012.2

[21] Schwingshackl P, Obermoser G, Nguyen VA, Fritsch P, Sepp N, et al (2012) Distribution and maturation of skin dendritic cell subsets in two forms of cutaneous T-cell lymphoma: mycosis fungoides and Sezary syndrome. Acta Derm Venereol 92: 269–275. 22678564 10.2340/00015555-1220

[22] Svajger U, Anderluh M, Jeras M, Obermajer N (2010) C-type lectin DC-SIGN: an adhesion, signalling and antigen-uptake molecule that guides dendritic cells in immunity. Cell Signal 22: 1397–1405. 20363321 10.1016/j.cellsig.2010.03.018 PMC7127357

[23] Cambi A, Gijzen K, de Vries l J, Torensma R, Joosten B, et al (2003) The C-type lectin DC-SIGN (CD209) is an antigen-uptake receptor for Candida albicans on dendritic cells. Eur J Immunol 33: 532–538. 12645952 10.1002/immu.200310029

[24] Sousa Mda G, Reid DM, Schweighoffer E, Tybulewicz V, Ruland J, et al (2011) Restoration of pattern recognition receptor costimulation to treat chromoblastomycosis, a chronic fungal infection of the skin. Cell Host Microbe 9: 436–443. 21575914 10.1016/j.chom.2011.04.005 PMC3098964

[25] Newman SL, Lemen W, Smulian AG (2011) Dendritic cells restrict the transformation of Histoplasma capsulatum conidia into yeasts. Med Mycol 49: 356–364. 21039309 10.3109/13693786.2010.531295

